# Summary of talks and papers at ISCB-Asia/SCCG 2012

**DOI:** 10.1186/1471-2164-14-S2-I1

**Published:** 2013-04-29

**Authors:** Konstantin Tretyakov, Tatyana Goldberg, Victor X Jin, Paul Horton

**Affiliations:** 1 Institute of Computer Science, University of Tartu, J. Liivi 2, 50409 Tartu, Estonia; 2 Faculty of Informatics, Department for Bioinformatics and Computational Biology, Technical University Munich, Boltzmannstrasse 3, Garching 85748, Germany; 3 Department of Biomedical Informatics, The Ohio State University, 460 W 12th Ave., 212 BRT, Columbus, OH 43210, USA; 4 Computational Biology Research Center, AIST, 2-4-7 Aomi, Koto-ku, Tokyo, 135-0064, Japan

## Abstract

The second ISCB-Asia conference of the International Society for Computational Biology took place December 17-19, 2012, in Shenzhen, China. The conference was co-hosted by BGI as the first Shenzhen Conference on Computational Genomics (SCCG).

45 talks were presented at ISCB-Asia/SCCG 2012. The topics covered included software tools, reproducible computing, next-generation sequencing data analysis, transcription and mRNA regulation, protein structure and function, cancer genomics and personalized medicine. Nine of the proceedings track talks are included as full papers in this supplement.

In this report we first give a short overview of the conference by listing some statistics and visualizing the talk abstracts as word clouds. Then we group the talks by topic and briefly summarize each one, providing references to related publications whenever possible. Finally, we close with a few comments on the success of this conference.

## Introduction

Following the success of the first ISCB-Asia, held jointly with APBioNET as InCoB/ISCB-Asia 2011 [1,2], ISCB-Asia/SCCG 2012 took place on December 17-19, 2012, in Shenzhen, China. This year BGI co-hosted ISCB-Asia as the first Shenzhen Conference on Computational Genomics (SCCG). ISCB-Asia/SCCG 2012 was immediately followed by the Asian Young Researchers Conference on Computational and Omics Biology (AYRCOB), also cohosted by BGI.

More than 146 people from more than 18 countries attended ISCB-Asia/SCCG 2012. The 45 conference talks included: 9 proceedings talks (selected from 26 submissions), 6 keynotes, 7 highlights, 3 technology track talks, 2 program chair-invited talks, and 4 special sessions (*Cancer genome informatics, Workflows and the cloud for reproducible bioinformatics, Computational statistics for modern biology, BGI special session*).

**Figure 1:**
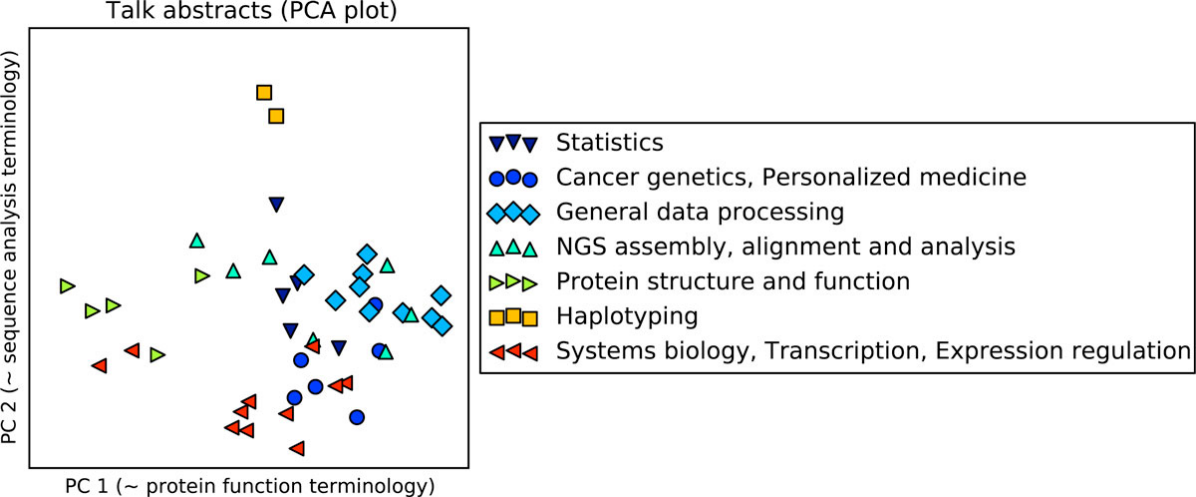
**Scatterplot visualization of conference talks**. A principal components scatterplot of conference talk abstracts. Each point represents a talk. Nearby points have many similar words in their abstracts. The principal component axes can be approximately interpreted as corresponding to the amount of "protein function" and "genomic sequence analysis"-related terminology.

**Figure 2:**
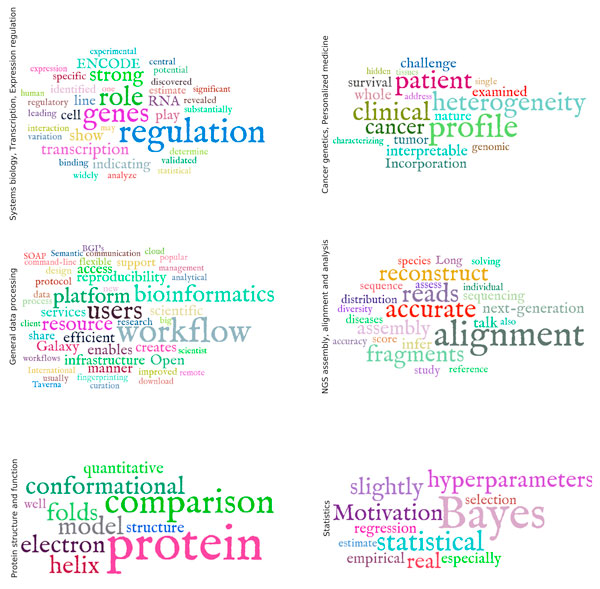
**Area-specific significant terminology**. Word clouds, illustrating terms specific for each of the major research areas covered in the conference. The size of each word is proportional to the overrepresentation log p-value of this term in the corresponding talk abstracts. P-values were computed by Fisher's exact test.

The talks were given by researchers from 16 countries, representing many of the leading centers of bioinformatics research worldwide, and the selection of topics was, in the opinion of the authors of this report, quite representative of modern-day trends in computational biology (Figures 1, 2).

This report briefly summarizes each talk given at the conference, grouped into six broad subject areas, ranging from data processing to statistics in modern biology.

## Data processing

As our community is struggling with the continuous deluge of "Big Data" [3], introduction of efficient tools and infrastructure for handling what are now petabyte-sized file collections, designing computational workflows and maintaining reproducible results becomes key for the future success of computational biology. Consequently, two sessions, organized by Scott Edmunds of *GigaScience*, were devoted to the topic of cloud-based tools, workflows and reproducible computing.

One focus area of ISCB-Asia/SCCG 2012 was workflow-management systems, with Galaxy [4-6] being one of the key platforms. *Genomic Data Submission and Analytical Platform (GDSAP) *[7] was presented by Tin-Lap Lee (Chinese University Hong Kong), as a CBIIT-led effort to provide a unified, Galaxy-based online toolkit for biomedical scientists. IRRI-Galaxy is a similar effort from the International Rice Research Institute (talk by Ramil Mauleon). Mohamed Abouelhoda (Nile and Cairo University) presented Tavaxy [8], another cloud-based system, which focuses on letting users combine and run workflows designed in both Galaxy and Taverna [9]. Finally, two commercial cloud-based data analysis systems were presented at the conference: *ClusterTech Life-science Analysis Suite (CLASS) *(tech talk by Ping Chung Ng) and BGI's *EasyGenomics *[10] (talk by Xu Xing).

While workflow systems allow researchers to efficiently design data analysis pipelines, they are not always successful at ensuring long-term reproducibility. More often than not, running the same workflow a year later would not yield the same results. This issue is addressed by the *Wf4Ever *project [11] presented by Marco Roos (Leiden University Medical Center), in which the idea of a *workflow *is generalized to the concept of a *research object*.

Reproducibility, interoperability and automation of workflows can be facilitated by the use of universally recognized metadata formats. The *ISA *metadata framework [12] aims to provide a set of such formats, standards and tools (talk by Eamonn Maguire, University of Oxford).

The issue of preserving privacy in the analysis of omics data is a growing concern [13]. In a technical talk, Kana Shimizu (AIST) described a clever application of additive homomorphic encryption which allows a database of chemical compounds to be interrogated for the presence or absence of records similar to a query compound - without revealing the query itself.

Finally, as detailed in article S8 of this supplement [14], Konstantin Tretyakov (University of Tartu) presented a new file fingerprinting method enabling fast synchronization of large biological data repositories within the cloud and between data centers.

## Sequence and NGS data analysis

Genomic sequence analysis techniques such as sequence alignment have long been considered a mature field and one of the cornerstones of computational biology. However, the rapid development of next-generation sequencing (NGS) technologies in the last decade continues to raise new unexpected challenges.

In meta-genomics, the genomes of all the organisms found in an environmental sample (e.g. soil) are pooled and sequenced together. The resulting genomic diversity severely complicates the task of NGS read assembly and introduces the related problem of *binning *reads by their source, where source is one or more closely related species. Francis Chin (The University of Hong Kong) presented Meta-IDBA [15] as a tool for solving both problems.

*De novo *assembly of RNA-seq (transcriptome) data also requires specialized algorithms and tools. Dongxiao Zhu (Wayne State University) presented SPATA [16], an RNA-seq assembly method based on a divide-and-conquer strategy.

Genome-wide methylation is often measured by applying NGS to bisulfite-converted DNA [17], in which unmethylated cytosines are converted to thymines. This conversion allows the position of methylated cytosines to be inferred by traditional sequence alignment procedures as described in a highlights talk by Martin Frith [18] (AIST).

An interesting method for performing phylogenetic analysis on NGS data without the need to assemble reads was presented in a highlights talk by Urmila Kulkarni-Kale [19] of the University of Pune.

Articles S6 and S7 in this supplement apply machine learning techniques to the problem of automatic detection and classification of non-coding RNAs. Kun Sun (The Chinese University of Hong Kong) presented iSeeRNA [20], a novel tool applying Support Vector Machine (SVM) classifiers to detect long intergenic non-coding RNAs (lincRNAs) from transcriptome sequence data. Meanwhile, Mark Menor (University of Hawaii) presented a state of the art multiclass classifier McRUM [21], evaluated on a dataset of small non-coding RNAs (small ncR-NAs).

Another central topic of NGS analysis is fast and accurate inference of genotypes. Articles S2 and S5 presented novel algorithms for haplotype reconstruction. Fei Deng (University of Hong Kong) presented a dynamic programming algorithm and heuristic speed-up for the minimum error correction formulation of this problem [22]. In a more probabilistic approach, Hirotaka Matsumoto (University of Tokyo) presented a mixture model based reconstruction method (MixSIH) and a new quality metric (MC) [23]. Meanwhile, in the special session on cloud computing, Junwen Wang (EasyGenomics, BGI) presented FaSD [24], a cloud based solution for fast and accurate detection of SNPs (Single Nucleotide Polymorphisms) from sequence data.

Finally, Ge Nong (Sun Yat-Sen University) gave an invited talk on efficient (linear-time) construction algorithms for suffix arrays [25], a data structure underlying many NGS applications.

## Systems biology, transcription and expression regulation

Elucidation of the mechanisms involved in gene expression, mRNA regulation and protein-protein interactions is among the central goals of contemporary biological research. Indeed, five out of the six conference keynote talks were largely related to this area of research. This field has recently gained a significant boost thanks to the completion of the ENCODE project [26], whose press releases attracted significant media attention -- in particular the claim that more than 80% of the human genome "works as a kind of control panel packed with genetic dials" [27], when previous conventional wisdom was that more than 95% of human DNA might be nonfunctional "junk".

The true significance of the often quoted 80% figure has, however, been widely debated [28]. According to the keynote speaker Philip Green (University of Washington), "junk DNA hasn't gone anywhere and is here to stay". In one of the most memorable moments of ISCB-Asia/SCCG 2012, Prof. Green eloquently challenged transcription (*i.e*. interaction with RNA-polymerase) as sufficient evidence of function, pointing out that although 100% of the genome interacts with DNA-polymerase (replication) we do not conclude that 100% of the genome is functional.

Nevertheless, no one doubts that transcription regulation is a vital and complex process, whose details remain unclear. A keynote by Piero Carninci (RIKEN) described studies using DeepCAGE [29], which enables highly sensitive detection of transcription start sites via NGS sequencing of 5' capped mRNAs. In a recent study, Carninci's group has used DeepCAGE to discover specific patterns of expression of retrotransposon elements in different cell compartments.

Methylation is one of the key mechanisms of mammalian gene regulation. In a keynote by Takashi Ito (University of Tokyo), a novel whole-genome bisulfite sequencing method was presented, which, in contrast to previous technologies, can be applied to minuscule quantities of material (only a few thousand cells) without the need for global PCR amplification.

Two talks focused on transcription factors and their binding. A keynote by Zhiping Weng (University of Massachusetts Medical School) introduced Factorbook [30], a *de novo *motif discovery analysis performed on ENCODE data; and Stephen Kwok-Wing Tsui (The Chinese University of Hong Kong) presented a method of discovering Protein-DNA binding sites using association rule mining [31].

miRNA was another topic addressed by multiple talks. As described in article S3 of this supplement [32], Toyofumi Fujiwara (INTEC) introduced a novel method for predicting miRNA targets, based on the hypothesis that promoters of miRNAs and their targets tend to share (predicted) cis-elements [32]. In a highlights talk, Michal Linial (The Hebrew University of Jerusalem) discussed a novel algorithm, miRror2.0, which enables the discovery of combinatorial regulation of transcription by several miRNAs [33].

Several talks introduced advances in the analysis of gene expression data. As detailed in article S9 [34], Koki Tsuyuzaki (Tokyo University of Science) proposed a novel information criterion based technique for detecting differentially expressed genes. Luonan Chen (Shanghai Institute for Biological Sciences) discussed the analysis of expression of the genes in the quiescence signaling pathways of *S. cerevisiae*. Finally, a keynote by Eric Xing (Carnegie Mellon University) presented TREEGL [35], an approach based on ℓ_1_-penalized linear regression, capable of reconstructing the evolution of a gene network from relatively scarce gene expression data.

We close this section by mentioning the highlights talk given by Mark Ragan (The University of Queensland), who reported an analysis of human transcriptomic data to elucidate the effect that alternative splicing has on the protein-protein interaction network, via inclusion or omission of certain interaction domains [36].

## Protein structure and function

The structure of a protein provides crucial insights into its functional role in a cell. Although the first protein structures were determined more than half a century ago, the structures of many proteins remained unsolved and the mechanism of protein folding is not yet fully understood.

In his keynote address, Gunnar von Heijne (Stockholm University) described quantitative analyses of the energetics and kinetics of membrane protein assembly *in vivo *that lead to a better understanding of the mechanisms of transmembrane protein assembly, and improvement of topology and structure-prediction methods. Many of these prediction methods sometimes confuse helical regions in membrane proteins with signal peptides in classically secreted proteins. In a highlights talk, Henrik Nielsen (Technical University of Denmark) described SignalP 4.0 [37], a new version of that popular software for predicting signal peptides. In addition to having a new architecture, SignalP now explicitly discriminates between signal peptides and transmembrane regions.

Superficially dissimilar protein sequences can adapt similar 3D structures and biological functions. Thus protein structure comparison methods are indispensable. Article S1 by Xiuzhen Huang et al. (Arkansas State University) describes ePC, an accurate and fast algorithm that is able to compare whole structures as well as specific substructures [38]. As detailed in article S4 [39], Prasad Gajula (Indian Agricultural Statistics Research Institute) presented a molecular dynamics simulation of the protein Vinculin and showed that the simulation is highly consistent with local mobility as determined experimentally by electron paramagnetic resonance (EPR) spectroscopy.

Two novel methods dealing with multimeric protein complexes were introduced by Daisuke Kihara (Purdue University): Multi-LZerD [40] for modeling the structure of protein complexes and EMLZ-erD [41] for fitting them into electron microscopy maps.

## Cancer genomics and personalized medicine

The special session on cancer research organized by Chen-Hsiang Yeang (Academica Sinica) focused on techniques for the integrative analysis of heterogeneous cancer data. Yinyin Yuan (Institute of Cancer Research) presented a quantitative image-based approach [42] that, in combination with molecular assays, is able to uncover new knowledge about breast tumor biology and predict patient survival. Robert Beckman (Daiichi Sankyo) introduced a computational model of targeted cancer therapy incorporating genetic evolutionary dynamics and single-cell heterogeneity [43]. He reported that in a large virtual clinical trial of cancer patients the model may lead to improved outcomes compared with the current personalized medicine approach. Shihua Zhang (Chinese Academy of Sciences) developed a method for the systematic analysis of multi-dimensional genomics data [44]. Using DNA methylation, gene expression and microRNA expression data of ovarian cancer samples, he showed that the method is able to uncover biologically relevant patterns of complex gene regulatory systems. Biaoyang Lin (Zhejiang-California International Nanosystems Institute) presented MAPS [45], a massive parallel sequencing method, to conduct a global survey of Hepatitis B virus (HBV) in HBV-related hepatocellular carcinomas (HCCs). The outcome of the survey contributed significantly to the understanding of the mechanism of HCC development.

In a technical track presentation, Frank Schacherer (BIOBASE GmbH) described Genome Trax and the Human Genome Mutation Database (HGMD) [46], two human-curated annotation sources for identifying functionally relevant variants in the human genome and understanding their effects in a medical context. HRRA and HaploShare [47] are two other sources for linking genetic variants with underlying disease phenotypes; presented by Wanling Yang (The University of Hong Kong), who also stressed the importance of making disease associations from exome or whole genome sequencing data easily interpretable for clinicians.

A tool for non-invasive prenatal diagnosis, FetalQuant [48], was presented in a highlights talk by Hao Sun (The Chinese University of Hong Kong). This tool estimates fractional fetal DNA concentration directly from massively parallel sequencing on DNA in maternal plasma, eliminating the need for prior genotype information.

## Statistics in modern biology

Recent advances in DNA chip technology and the discovery of thousands of SNPs in genome-sequencing projects motivated genomic selection using high-density markers. However, the increasing number of available biomarkers presents both computational and statistical challenges. The special session on Statistics in Modern Biology organized by Dabao Zhang (Purdue University) addressed both issues.

Vitara Pungpapong (Chulalongkorn University) proposed a fast and accurate algorithm for biomarker selection, implementing an empirical Bayes method for variable selection in regression models [49]. POCRE, the penalized orthogonal-components regression method [50], is another predictor for genomic selection, presented by Min Zhang (Purdue University). It outperforms its competitor BayesB [51] in both time and performance at estimating breeding values. Yuanhui Xiao (Georgia State University) presented analysis of retinal pigment epithelium flatmount images.

## Conclusions

We subjectively conclude that ISCB-Asia/SCCG 2012 was without doubt a successful event. At least three continents (Asia, Europe & North America) and all age groups were well represented, but there was naturally a strong contingent of Asians and young people, which we believe bodes well for the future of computational biology in Asia. Indeed, The Asian Young Researchers Conference on Computational and Omics Biology (AYRCOB), also co-hosted by BGI, immediately followed ISCB-Asia/SCCG 2012 in Shenzhen and effectively served as an extension of ISCB-Asia/SCCG 2012 for student participants. Finally we note that the 23rd Annual Genome Informatics Workshop held in Tainan, Taiwan during the week previous to ISCB-Asia/SCCG 2012 was also highly successful, enjoying a record number of paper submissions.

Among other memorable aspects of ISCB-Asia/SCCG 2012 were the social events organized after the long days of presentations and poster sessions. The quiet get-togethers in the nearby Chinese pubs and the organized excursion to BGI, followed by a dinner, all were great opportunities for the speakers and other participants of the meeting to come together in an informal environment and participate in relaxed and open discussions. The rather compact size of the conference added an exceptional degree of friendliness. Established researchers seemed to be much more accessible to graduate students than it is usually the case in larger conferences.

## Competing interests

KT is a coauthor of article S8 of this supplement and mentioned in this report. KT and TG gave talks at the conference. VJ was program committee chair. PH was conference chair.

## Authors' contributions

KT, TG contributed equally to writing the manuscript. KT prepared the figures. PH edited an initial draft of the manuscript. All authors read and approved the final manuscript.

## Acknowledgements

We would like to acknowledge BGI for co-hosting the conference and the conference sponsors BIOBASE, CLUSTERTECH and AIST for financial support. Huinan Hao and the rest of the BGI staff for excellent ground support during the conference. The Kingkey Palace hotel staff for their cooperation and delicious lunches. The ISCB management: in particular B.J. Morrison for her time and care, Janet Kelso for her encouragement, and Burkhard Rost, Rheinhard Schneider and Michal Linial, who traveled great distance to support the conference. We thank all members of the conference advisory committee, steering committee, and program committee and the anonymous volunteers who served to review procedings and highlights track submissions. We thank the keynote speakers, who took time out of their busy schedules to give six excellent talks. Last but not least, we thank Catherine Wells and the BMC editors for their help in publishing this supplement.

## Declarations

The publication costs for this article were funded by general reseach funds of the National Institute of Advanced Industrial Science and Technology (AIST), Japan.

This article will be published as the introduction to *BMC Genomics *Volume 14 Supplement 2, 2013: Selected articles from ISCB-Asia 2012. The full contents of the supplement are available online at http://www.biomedcentral.com/bmcgenomics/supplements/14/S2.
